# Comparative evaluation of rapid tests, HIV specific peptide EIA and Western Blot test for diagnosis of HIV-2 and HIV 1&2 infection

**DOI:** 10.1186/1471-2334-12-S1-P20

**Published:** 2012-05-04

**Authors:** Jayanthi Shastri, Sachee Agrawal, Sandhya Sawant, Manish Pathak, Bharat Parekh

**Affiliations:** 1 Department of Microbiology, Topiwala National Medical College & B. Y. L. Nair Ch. Hospital, Mumbai, India; 2 Centers for Disease Control & Prevention, Atlanta, USA

## Background

In 1986, a second type of HIV, called HIV-2, was isolated from AIDS patients in West Africa, where it may have been present decades earlier. HIV-2 infections are predominantly found in Africa. West African nations report a prevalence of HIV-2 infection of more than 1% in the general population. The prevalence rate of HIV-2 infection in India is not available so far as HIV-2 infections are underreported in India. The currently used Rapid Antibody diagnostic tests when used singly are unable to specifically detect HIV-2 and HIV 1&2 infections. Hence the present study was undertaken to specifically detect HIV-2 and HIV 1&2 infections and validate the currently used Rapid test kits using HIV specific peptide EIA and Western Blot test for diagnosis of HIV-2 and HIV 1&2 infection.

## Methods

A total of 37 serum samples (18 HIV-2 and 19 HIV 1&2) were evaluated by using HIV specific peptide EIA (CDC Atlanta, USA) and Western Blot test. All these 37 samples were diagnosed HIV-2 and HIV 1&2 as per the guidelines laid down by the National AIDS Control Organization (NACO), India.

## Results

100% concordance was observed in the results of Rapid tests, EIA test and Western Blot test.

## Conclusion

Under all circumstances screening for diagnosis of HIV should be based on three different tests with three different antigenic principles. Using above protocol it is possible to detect HIV-2 and HIV 1&2 infections.

